# Frequent downregulation of 14-3-3 *σ* protein and hypermethylation of *14-3-3 σ* gene in salivary gland adenoid cystic carcinoma

**DOI:** 10.1038/sj.bjc.6602004

**Published:** 2004-08-03

**Authors:** D Uchida, N-M Begum, A Almofti, H Kawamata, H Yoshida, M Sato

**Affiliations:** 1 Second Department of Oral and Maxillofacial Surgery, Tokushima University School of Dentistry, 3-18-15 Kuramoto, Tokushima 770-8504, Japan; 2 Department of Surgical and Molecular Pathology, Dokkyo University School of Medicine, 880 Kita-kobayashi, Mibu, Shimo-tsuga, Tochigi 321-0293, Japan

**Keywords:** adenoid cystic carcinoma, mucoepidermoid carcinoma, 14-3-3 *σ*, p53, methylation

## Abstract

*14-3-3 σ*, a target gene of the p53 tumour suppressor protein, has been shown to regulate the cell cycle at the G2/M checkpoint. Recent studies have demonstrated that 14-3-3 *σ* is downregulated by hypermethylation of the CpG island in several types of cancer. In this study, we investigated the expression and methylation status of *14-3-3 σ* in human salivary gland adenoid cystic carcinoma (ACC) and mucoepidermoid carcinoma (MEC). Immunohistochemical analysis revealed that the positive expression rate of 14-3-3 *σ* in ACC (one out of 14) was markedly lower than that in MEC (ten out of 10). Since most of the ACCs carried the wild-type p53 protein, downregulation of 14-3-3 *σ* in ACC may not be due to the dysfunction of p53 pathway. Microdissection–methylation-specific PCR revealed that frequent hypermethylation of the *14-3-3 σ* gene was observed in ACC when compared to that in MEC. In cultured-ACC cells, we confirmed the downregulation of 14-3-3 *σ* via hemimethylation of the gene by sequencing analysis after sodium bisulphite treatment. Furthermore, re-expression of 14-3-3 *σ* in the ACC cells was induced by the treatment with DNA demethylating agent, 5-aza-2′-deoxycytidine. Irradiation apparently induced the enhanced expression of 14-3-3 *σ* and G2/M arrest in normal salivary gland cells; however, in the ACC cells, neither induction of 14-3-3 *σ* nor G2/M arrest was induced by irradiation. These results suggest that downregulation of 14-3-3 *σ* might play critical roles in the neoplastic development and radiosensitivity of ACC.

Salivary gland cancers (SGCs) are somewhat rare diseases, comprising about 10% of the head and neck cancers (Speight and Barrett, 2002). Estimated annual incidence of this diseases is approximately 400 cases in Japan (Ajiki *et al*, 2001); however, it is well known that prognosis of SGCs is considerably poor (Bensadoun *et al*, 2001). Salivary gland cancers are divided histologically into low-grade and high-grade malignancies (Seifert and Sobin, 1992). High-grade SGCs, such as adenoid cystic carcinoma (ACC) and mucoepidermoid carcinoma (MEC) (Batsakis, 1979), invade the surrounding tissues (including nerve, muscle, and cutaneous tissue), and frequently metastasise to the regional lymph nodes and distant organs, such as lungs and brain. Adenoid cystic carcinoma and MEC, the most common malignancies of the salivary glands, are clinically and pathologically different. Adenoid cystic carcinoma occurs frequently in the minor salivary glands (59% of total malignant tumours in minor salivary gland; Le *et al*, 1999). On the other hands, MEC occurs frequently in the major salivary glands (51% of total malignant tumours in major salivary gland; Pinkston and Cole, 1999). Although radical surgery has been the main therapy for these cancers, irradiation has often been recommended for the treatment of the advanced SGCs. However, the radiosensitivity is reported to be quite different in ACC and MEC. Combination radical surgery with radiotherapy in ACC has led to superior results in many studies (Simpson *et al*, 1984; Vikram *et al*, 1984; Miglianico *et al*, 1987; Garden *et al*, 1995; Parsons *et al*, 1996), but in MEC such combination treatment is generally less effective (Nascimento *et al*, 1986; Grenman *et al*, 1992). These findings suggest that cell cycle-related gene(s) at the G2 checkpoint might play important roles in the difference in radiosensitivity between these cancers, since the G2-residing cells are normally more sensitive to irradiation. However, alteration of G2 cell cycle regulators such as p53 is reported to be infrequent in SGCs (Papadaki *et al*, 1996; Tsubochi *et al*, 2000; Kiyoshima *et al*, 2001). Thus, it is possible that novel G2 cell cycle regulator(s) contribute to the different radiosensitivity in SGCs.

*14-3-3 σ* (also called stratifin) was first identified as a gene expressed specifically in the differentiated squamous epithelium, and loss of 14-3-3 *σ* immortalised squamous epithelium (Leffers *et al*, 1993). A subsequent study showed that 14-3-3 *σ* was strongly induced by *γ*-irradiation and other DNA-damaging agents via a p53-dependent pathway (Hermeking *et al*, 1997). Moreover, Chan *et al* (1999) reported that 14-3-3 *σ*-deficient cells were rapidly killed by ionizing radiation through a process known as mitotic catastrophe, which was associated with a failure of the cells to sequester cyclin B/CDC2 complex from the nucleus. More recently, it was shown that the expression of 14-3-3 *σ* was frequently lost in several types of cancers due to hypermethylation of the gene (Ferguson *et al*, 2000; Iwata *et al*, 2000; Suzuki *et al*, 2000; Gasco *et al*, 2002a, 2002b; Osada *et al*, 2002). These findings indicate that 14-3-3 *σ* plays diverse roles not only in the radiosensitivity of the cells but also in tumorigenesis of several cancer, including SGCs.

In this study, we investigated the expression of 14-3-3 *σ* protein and methylation status of *14-3-3 σ* gene in ACC and MEC. Then, we report for the first time that 14-3-3 *σ* in salivary gland ACC is frequently downregulated due to the hypermethylation of the gene.

## MATERIALS AND METHODS

### Immunohistochemistry (IHC)

Salivary gland cancer tissues surgically obtained at the Tokushima University School of Dentistry from 1982 to 2002 were routinely fixed with formalin and embedded in paraffin. Before staining, sections 4 *μ*m thick mounted on poly-L-lysine-coated slides were deparaffinised with xylene and rehydrated in graded ethanol. For the purpose of antigen retrieval, sections were incubated in Target retrieval solution (DAKO, Carpinteria, CA, USA) at 95°C for 20 min. After blocking with 3% horse serum in phosphate-buffered saline, samples were incubated at room temperature for 1 h with the goat polyclonal 14-3-3 *σ* antibody (Santa Cruz Biotechnology, Santa Cruz, CA, USA) diluted to 1 : 100 and monoclonal p53 antibody (DO-7; DAKO) diluted to 1 : 100 with the blocking solution, and for the subsequent steps the avidin–biotin–peroxidase method was performed using a Vectastain ABC kit (Vector, Burlingame, CA, USA). We defined the cases, in which over 25% of the cancer cells were stained for 14-3-3 *σ* or p53, as IHC-positive (+). When the ratio of the positive-stained cells did not reach 25%, or the cells were not stained for 14-3-3 *σ* or p53, we defined these cases as IHC-negative (−).

### Methylation-specific PCR (MSP)

The cancer nests in the five 10 *μ*m-thick paraffin sections were dissected under the stereomicroscopy with a 24-gauge needle and mineral oil. DNA was prepared using an EX-WAX DNA Extraction kit (Serologicals Corporation, Norcross, GA, USA) according to the manufacturer's instructions. In all, 1 *μ*g of genomic DNA was subjected to sodium bisulphite modification by a CpGenome DNA modification kit (Serologicals Corporation), and then MSP was performed by using a primer set that covered four CG dinucleotides of *14-3-3 σ* DNA as described previously (Ferguson *et al*, 2000). For the *p16*^*INK4A*^ gene, PCR was performed under the same conditions as described by Herman *et al* (1996). Densitometric analysis of the data was carried out by NIH imaging software (ver. 1.63). Methylation index was calculated as follows: the density of methylated band was divided by that of unmethylated band.

### Cell lines and culture

Adenoid cystic carcinoma cell lines, ACCS and ACCY (Shirasuna *et al*, 1990), were kindly provided by Drs Hiroaki Ishibashi and Kanemitsu Shirasuna (Second Department of Oral and Maxillofacial Surgery, Kyusyu University School of Dentistry). Normal salivary gland cells (SG1 and SG2) were independently cultured from normal human submandibular gland tissue. TYS (Yanagawa *et al*, 1986) and HSG (Shirasuna *et al*, 1981) were salivary adenosquamous cell carcinoma cells and neoplastic human salivary intercalated duct cells, respectively. All the cells except normal salivary gland cells were maintained in DMEM supplemented with 10% FCS, 100 *μ*g ml^−1^ streptomycin, and 100 U ml^−1^ penicillin in a humidified atmosphere of 95% air and 5% CO_2_ at 37°C. Salivary gland cells were maintained in Keratinocyte-SFM (Invitrogen, Carlsbad, CA, USA) and antibiotics.

### PCR–single-strand conformation polymorphism (SSCP)

Exons 5–8 of the *p53* gene were examined for mutations by PCR–SSCP analysis. Genomic DNA was amplified by PCR with four sets of primers. Primers for exon 5 were as follows: 5R, 5′-ACCCTGGGCAACCAGCCCTG-3′ (sense); 5L, 5′-TTTATCTGTTCACTTGTGCCC-3′ (antisense). Primers for exon 6 were 6R, 5′-CTCCCAGAGACCCCAGTTGC-3′ (sense); 6L, 5′-TCAGATAGCGATGTGAGCAG-3′ (anti-sense). Primers for exon 7 were 7R, 5′-CAGTGTGCAGGGTGGCAAGT-3′ (sense); 7L, 5′-GCCACAGGTCTCCCCAAGGC-3′ (anti-sence). Primers for exon 8 were 8R, 5′-CCACCGCTTCTTGTCCTGCT-3′ (sense); 8L, 5′-GACCTGATTTCCTTACTGCC-3′ (anti-sense). Each target sequence was amplified in a 20 *μ*l reaction volume containing 100 ng genomic DNA, 0.2 *μ*M dNTPs, 0.5 *μ*M of each primer and 0.05 U *μ*l^−1^ Advantage DNA polymerase (BD Clontech, Palo Alto, CA, USA) using the hot-start method. PCR amplification consisted of an initial activation step of 95°C for 3 min, followed by 40 cycles of 95°C for 1 min, 56 or 58°C for 1 min, and 72°C for 4 min. PCR products were diluted two-fold with formamide dye solution, denatured at 95°C for 5 min and separated by electrophoresis on 12.5% polyacrylamide gels with 5% glycerol at constant temperature (14°C). Gels were stained and visualised with a Silver Staining II kit (Bio-Rad Laboratories, Hercules, CA, USA).

### RNA preparation and Northern blot analysis

Cells (ACCS, ACCY, TYS, HSG, and SG) grown in monolayers were harvested at early confluence. After 24 h, RNA isolation was performed using TRIzol reagent (Invitrogen) according to manufacture's instructions. RT–PCR and Northern blotting were carried out as described previously (Kawamata *et al*, 1998). The probes consisted of a 278 bp fragment of human *14-3-3 σ* cDNA amplified from SG cDNA. PCR for mutation analysis of *p53* mRNA was carried out under the following conditions: 95°C for 3 min, followed by 30 cycles of 94°C for 1 min, 62°C for 45 s, and 72°C for 1 min, and a final extension at 72°C for 10 min. Amplified fragments for the *p53* open reading frame were cloned by a TOPO TA cloning system (Invitrogen), and five independent clones were sequenced by an ALOKA DNA sequencer (ALOKA, Tokyo, Japan). The primers used were as follows: for 14-3-3 *σ*, 5′-GTGTGTCCCCAGAGCCATGG-3′ and 5′-ACCTTCTCCCGGTACTCACG-3′; for p53, 5′-GGAATTCCGATGGAGGAGCCGCAGTCAG-3′ and 5′-GCGTCGACTCAGTCTGAGTCAGGCCCTTC-3′.

### Western blotting

Cells were seeded at a density of 1 × 10^5^ cells per 60 mm culture dish. Next, the cells were lysed with a buffer containing 50 mM Tris-HCl (pH 7.5), 150 mM NaCl, 1% Nonidet P-40, and a protease inhibitor cocktail tablet (Roche Molecular Biochemicals, Mannheim, Germany). After centrifugation, aliquots of the supernatant containing 30 *μ*g protein were analysed by 10% SDS–polyacrylamide gel electrophoresis. The separated proteins were transferred to a polyvinylidene difluoride membrane (Amersham Pharmacia Biotech., Uppsala, Sweden), and the membrane was incubated with primary antibodies against 14-3-3 *σ*, and *β*-actin (Sigma, St Louis, MO, USA), followed by horseradish peroxidase-conjugated secondary antibodies. Detection was then performed using an enhanced chemiluminescence kit (Amersham Pharmacia Biotech.).

### Sequencing analysis of 14-3-3 *σ* gene after sodium bisulphite treatment

Genomic DNA was prepared by the SDS–proteinase K method, and then subjected to the sodium bisulphite modification. The DNA modification was performed by a CpGenome DNA modification kit (Serologicals Corporation) as described above. Bisulphite-converted DNA was amplified, using primers that generated a 474-bp PCR product, as described previously (Ferguson *et al*, 2000). Amplified PCR products were cloned by using the TOPO TA cloning system, then DNA sequencing was performed using an ALOKA DNA sequencer. Five independent clones were sequenced for each cell line.

### Treatment of cells with 5-aza-2′-deoxycytidine

Cells were seeded at a density of 2 × 10^6^ cells 60 mm plate^−1^. After 24 h, cells were treated with or without 5 *μ*M 5-aza-2′-deoxycytidine (Sigma) for 3 days. Preparation of total RNA, protein, and RT–PCR were performed under the conditions as described above.

### Irradiation of the cells

Logarithmically growing cells (SG1 and ACCY) were exposed to X-ray irradiation at 15 Gy (150 kVp, 5 mA; MBR-1505R2, Hitachi Medico, Tokyo, Japan). After 24, 48, and 72 h, the cells were harvested, and then the cells were subjected to flow cytometric analysis or Western blotting.

### Flow cytometric analysis

After irradiation, the cells were trypsinised and fixed with 70% cold ethanol. Then, the precipitates were incubated with propidium iodide (PI) (40 *μ*g ml^−1^) and RNaseA (100 *μ*g ml^−1^) for 30 min at 37°C, and analysed with an EPICS flow cytometer (Coulter, San Jose, CA, USA).

## RESULTS

### Expression of 14-3-3 *σ* in SGCs

**Figure 1:**
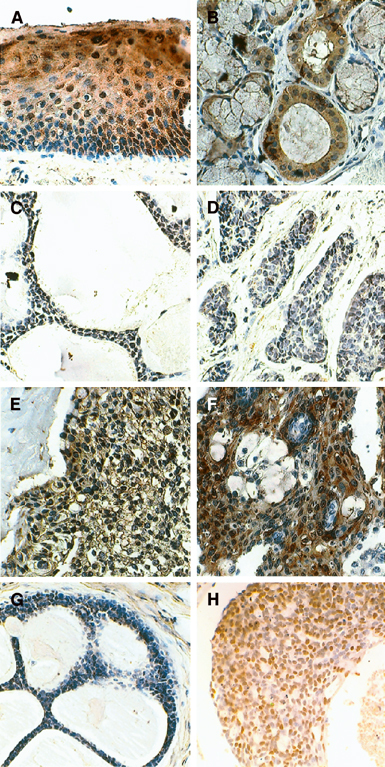
Immunohistochemical detection of 14-3-3 *σ* (**A–F**) and p53 (**G, H**). (**A**) Normal gingiva. (**B**) Normal salivary gland. (**C**) Adenoid cystic carcinoma with cribriform subtype. (**D**) Adenoid cystic carcinoma with solid subtype. (**E, F**) Mucoepidermoid carcinoma. (**G**) Adenoid cystic carcinoma with negative staining of p53. (**H**) Adenoid cystic carcinoma with positive staining of p53. Original magnification, × 400.

We examined the expression of 14-3-3 *σ* in 10 cases of normal salivary gland, 14 cases of ACC, and 10 cases of MEC. In several SGCs occurred at minor salivary gland, we could observe the normal gingival tissue in the surgically resected samples. We used normal gingival tissue as positive control for IHC of 14-3-3 *σ*. In normal gingival epithelial cells, 14-3-3 *σ* was barely detectable in the basal layer, but was increased in the suprabasal layers (Figure 1A

), as described by Dellambra *et al* (2000). In normal salivary gland, we found the positive staining of 14-3-3 *σ* protein on the salivary duct cells and the mucous gland cells, but not in the myoethithelial cells (Figure 1B). In ACCs, expression of 14-3-3 *σ* was hardly detected in the neoplastic myoepihtelial cells, but faintly in the neoplastic duct cells (Figure 1C and D). Only in one case of 11 ACCs, more than 20% of the cells (mainly in the neoplastic duct cells) were positive for 14-3-3 *σ* (Figure was not shown, Table 1

**Table 1:** Summary of the IHC, MSP, and PCR–SSCP

		**IHC**		**Methylation index**		**PCR–SSCP**
**No.**	**Primary site**	**14-3-3 *σ***	**p53**	**14-3-3 *σ***	**p16**	**p53 (exons 5–8)**
ACC1	Palate	−	+ (N)	2.25	0.29	Exon 7
ACC2	Submandibular gland	−	−	1.40	0.31	NC
ACC3	Floor of mouth	−	+ (N)	0.45	0.31	Exon 5
ACC4	Floor of mouth	−	−	3.26	1.75	Exon 6
ACC5	Tongue	−	−	0.59	0.14	NC
ACC6	Maxillary sinus	−	−	0.78	1.35	NC
ACC7	Palate	−	−	0.96	0.88	NC
ACC8	Sublingual gland	−	−	0.85	1.07	Exon 6
ACC9	Mandible	−	−	ND	ND	NC
ACC10	Floor of mouth	+	−	ND	ND	NC
ACC11	Palate	−	−	ND	ND	ND
ACC12	Palate	−	−	ND	ND	ND
ACC13	Maxillary sinus	−	−	ND	ND	ND
ACC14	Submandbular gland	−	−	ND	ND	ND
						
Mean				1.32	0.76	
Positive		1	2			
						
MEC1	Palate	+	−	0.72	0.24	NC
MEC2	Buccal mucosa	+	+ (C)	0.41	0.37	Exons 5–7^a^
MEC3	Buccal mucosa	+	−	0.56	2.30	NC
MEC4	Mandible	+	−	0.11	0.67	NC
MEC5	Palate	+	+ (C)	0.80	0.11	Exon 6
MEC6	Palate	+	−	0.44	0.21	NC
MEC7	Maxillary sinus	+	−	0.41	0.27	NC
MEC8	Parotid gland	+	−	0.34	0.25	ND
MEC9	Mandible	+	−	ND	ND	ND
MEC10	Submandibular gland	+	−	ND	ND	ND
						
Mean				0.47	0.55	
Positive		10	2			

−=negative staining; +=positive staining; ND=not determined; (N)=staining in the nucleus; (C)=staining in the cytoplasm. Methylation index was calculated as follows: the density of methylated band was divided by that of unmethylated band. NC=no change; ND=not determined; IHC=immunohistochemistry; MSP=methylation-specific PCR; PCR-SSCP=PCR–single-strand conformation polymorphism.

a Not amplified.

). However, in MECs, most of the cells in most of the cases showed positive staining for 14-3-3 *σ* (Figure 1E and F). We summarised the result of the IHC for 14-3-3 *σ* in Table 1.

### Mutational status of p53 and the relation of the expression pattern of 14-3-3 *σ* with the p53 status in SGCs

14-3-3 *σ* is also known as a cell cycle regulator of the G2 phase, and is a target gene of the tumour suppressor p53 protein. Therefore, mutational status of p53 in ACC and MEC was examined by IHC and PCR–SSCP. Immunohistochemical study revealed that strong staining of p53 protein was infrequent both in ACC (three out of 14) and MEC (two out of 10) (Figure 1G, H and Table 1). Moreover, as shown in Table 1, PCR–SSCP revealed that the mutation rate of the *p53* gene was increased – but still relatively low – in both ACC and MEC. There are no clear relationship between the expression pattern of 14-3-3 *σ* and p53 status in both ACC and MEC (Table 1).

### Methylation status of *14-3-3 σ* gene and *p16INK4A* gene in SGCs

**Figure 2:**
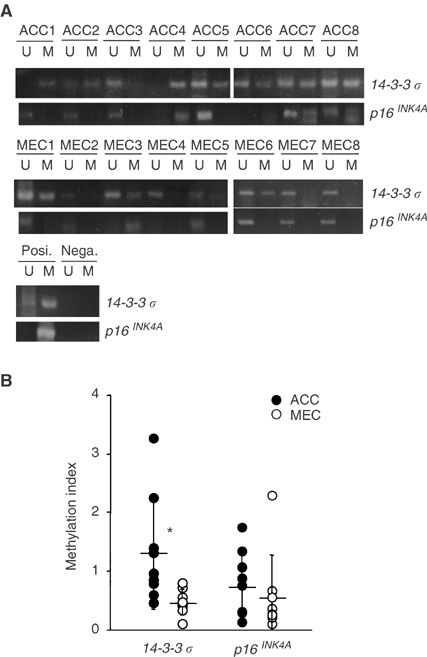
Methylation-specific PCR of the CpG island of the *14-3-3 σ* and *p16*^*INK4A*^ genes in ACC and MEC. (**A**) Microdissected tissues were subjected to MSP, and electrophoresis was performed in 4% agarose gels. The human fibrosarcoma cell line HT1080 was used as a positive control for methylated *14-3-3 σ*. Normal salivary gland cells (SG) treated with SssI methyltransferase were used as a positive control for *p16*^*INK4A*^. Water was used as a negative control. (**B**) Methylation of the *14-3-3 σ* and *p16*^*INK4A*^ genes was semiquantitated by densitometric analysis. Methylation indices were calculated by dividing the density of the unmethylated allele into that of the methylated allele. Horizontal bars and vertical bars show mean and s.d., respectively. ^*^Statistically significant by one-way ANOVA (^*^P=0.0304).

It has been reported that downregulation of 14-3-3 *σ* in several cancers was due to hypermethylation of the gene. Therefore, methylation status of the *14-3-3 σ* gene in ACC and MEC was examined by Microdissection–MSP method. The *14-3-3 σ* gene was frequently hypermethylated in ACC, but not in MEC (Figure 2A

). In addition, densitometric analysis revealed that the methylation index of ACC was significantly higher than that of MEC (one-way ANOVA, *P*=0.0304; Figure 2B). We also examined the methylation status of the *p16*^*INK4A*^ gene, which has been shown to be frequently hypermethylated in several types of cancers (Herman *et al*, 1996; Rocco and Sidransky, 2001), and was coincidentally inactivated with 14-3-3 *σ* in oral and vulval squamous cell carcinoma (Gasco *et al*, 2002a, 2002b). The methylation of *p16INK4A* was less frequent than that of *14-3-3 σ* in ACC, but was infrequently detected both in these cancers at the same level (Figure 2A, B).

### Expression of 14-3-3 *σ* in cultured-human SGC cells

**Figure 3:**
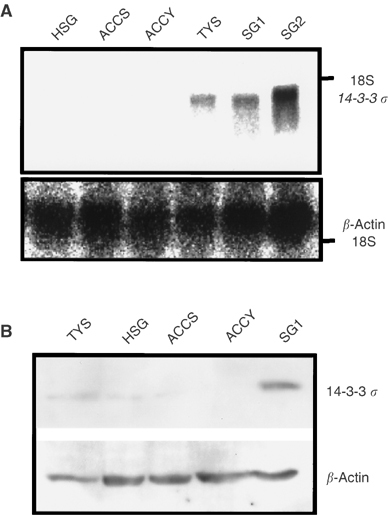
Expression of 14-3-3 *σ* in human SGC cells. (**A**) Detection of *14-3-3 σ* mRNA by Northern blot analysis. (**B**) Detection of 14-3-3 *σ* protein by Western blot analysis.

To confirm the result from the clinical materials, we examined the expression of 14-3-3 *σ* in cultured-human SGC cells. By means of Northern (Figure 3A

) and Western blotting (Figure 3B), downregulation of *14-3-3 σ* was detectable in two human ACC cell lines, ACCS and ACCY, and a neoplastic human salivary intercalated duct cell line, HSG, in comparison with a human salivary adenosquamous carcinoma cell line, TYS, and primary cultured-normal salivary gland cells, SGs.

### Epigenetic change of *14-3-3 σ* gene in cultured-ACC cells

**Figure 4:**
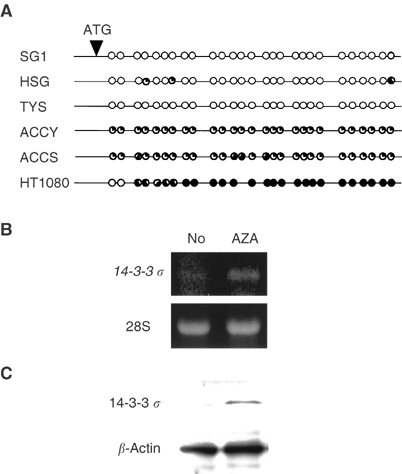
Methylation status of the *14-3-3 σ* gene in human SGC cells. (**A**) Sodium bisulphite sequencing of the *14-3-3 σ* CpG island. The methylated CpG sites are indicated by solid circles, and the unmethylated CpG sites are indicated by open circles. The cell lines examined are shown on the left. (**B**) Reactivation of *14-3-3 σ* mRNA in ACCY cells by treatment with 5-aza-2′-deoxycytidine for 3 days. RT–PCR was performed by use of specific primers for 14-3-3 *σ*. The 28S ribosomal RNA are shown below. (**C**) Reactivation of *14-3-3 σ* protein in ACCY cells by treatment with 5-aza-2′-deoxycytidine for 5 days. Western blotting was performed by use of specific antibodies for 14-3-3 *σ* and actin.

In order to clarify the mechanism for the downregulation of 14-3-3 *σ* in cultured-ACC cell lines, the methylation status of *14-3-3 σ* gene was assessed by the sequencing analysis after treatment with sodium bisulphite. As shown in Figure 4A

, normal salivary gland cells, SG1, and a human adenosquamous carcinoma cell lines, TYS, showed no methylation in *14-3-3 σ* gene, and a neoplastic human salivary intercalated duct cell line, HSG, showed very limited hemimethylation pattern in *14-3-3 σ* gene. However, the ACC cell lines, ACCS and ACCY, carried widely hemimethylated *14-3-3 σ* DNA (Figure 4A). In the human fibrosarcoma cell line, HT1080 we used as positive control (Leffers *et al*, 1993), *14-3-3* gene was almost completely methylated (Figure 4A). We further confirmed the association between the methylation pattern and downregulation of 14-3-3 *σ* by using a DNA demethylating agent, 5-aza-2’-deoxycytidine. Treatment of ACCY cells with 5-aza-2′-deoxycytidine at 5 *μ*M clearly upregulated the *14-3-3 σ* mRNA and protein (Figure 4B, C).

### Impaired G2/M arrest in ACC cells

**Figure 5:**
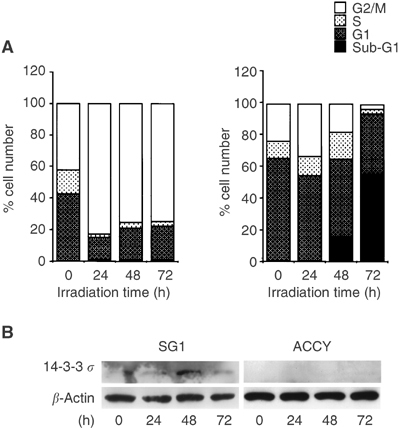
Cell cycle analysis of the SGC and normal cells after irradiation. (**A**) Cells were irradiated at 15 Gy, then harvested at 24, 48, and 72 h after irradiation. After staining with PI, cell cycles were analysed by flow cytometry. The populations of each phase are shown for the SG1 cells (left) and ACC cells (right). (**B**) Expression of the 14-3-3 *σ* protein after irradiation.

As shown in Figure 5A and B

, accumulation of the cells in the G2/M phase and strong induction of the 14-3-3 *σ* protein after irradiation were observed in the normal salivary gland cells, SG1. In contrast, irradiation did not induce the expression of 14-3-3 *σ* and G2/M arrest in ACCY cells, but markedly increased the cell population at the sub-G1 peak, indicating cell death.

## DISCUSSION

In this study, we examined the expression of 14-3-3 *σ* in the most common human SGCs, ACC and MEC. The findings obtained from the present series of experiments are as follows. First, expression of 14-3-3 *σ* was frequently lost in ACC, but not in MEC. Second, downregulation of 14-3-3 *σ* in ACC was not due to the alteration of the p53 tumour suppressor protein, but rather to hypermethylation of the CpG islands of *14-3-3 σ* gene. Third, a cultured-ACC cell line, ACCY, also showed downregulation of 14-3-3 *σ* and hypermethylation of the CpG islands of *14-3-3 σ* gene. Fourth, 14-3-3 *σ* was not induced by irradiation in ACCY, and ACCY was very sensitive to the irradiation. These results suggest that downregulation of 14-3-3 *σ* might be a critical event not only for the development of salivary gland ACC but also for the radiosensitivity of ACC cells.

Salivary gland ACCs are histologically divided into three groups: cribriform, tubular, and solid subtypes. Adenoid cystic carcinoma with solid subtypes has shown the worst prognosis. Stallmach *et al* (2002) reported that loss of heterozygosity at chromosome 6q23–25 was frequently found in the histological solid subtype of ACC. Yamamoto *et al* (1996) also demonstrated that the loss of heterozygosity in the p53 gene was higher in the solid subtype than in the cribriform subtype. In our present experiment, downregulation of 14-3-3 *σ* frequently occurred in various subtypes of ACC, but not in MEC. In normal salivary gland, 14-3-3 *σ* was highly expressed in duct cells and acinic cells but faintly in the myoepithelial cells. Mucoepidermoid carcinoma composed of the neoplastic epidermoid cells probably originated from precursor duct cells and of the neoplastic mucous producing cells, but most of the ACC are predominantly composed of the neoplastic myoepithelial cells. Thus, if the expression of 14-3-3 *σ* might be downregulated even in the neoplastic myoepithelial cells, then the overall expression of 14-3-3 *σ* in ACC was markedly limited.

It was reported that coincident epigenetic silencing of 14-3-3 *σ* and p16^INK4A^ by methylation was an early event of carcinogenesis in oral cancers and vulval SCC (Gasco *et al*, 2002a, 2002b). Moreover, downregulation of 14-3-3 *σ* was reported to allow primary human keratinocyte to escape replicative senescence accompanied by downregulation of p16^INK4A^ (Dellambra *et al*, 2000). In our present study, we could detect frequent hypermethylation of *14-3-3 σ* gene in ACC, but not that of *p16*^*INK4A*^ gene. However, three ACCs (ACC4, 6, 9), which have comparatively high methylation index of p16^INK4A^, showed histopathologically solid subtypes. Interestingly, Shintani *et al* (2000) have reported that p16^INK4A^ absent cases were the tumours with predominantly solid pattern by means of immunohistochemistry. Thus, in salivary gland ACC, downregulation of p16^INK4A^ might be critical event for the acquisition of histological subtype of ACC, and epigenetic silencing of 14-3-3 *σ* was also considered to be an early event of carcinogenesis. Then, the genetic changes, such as *p53* mutation, may occur in the subsequent step on the salivary gland carcinogenesis. Osada *et al* (2002) demonstrated that small-cell lung cancer cell lines frequently showed DNA hypermethylation of *14-3-3 σ*, but non-small-cell lung cancer cell lines did not. In our experiment, methylation pattern was quite different in the histological subtypes of SGCs, ACC, and MEC. Thus, methylation pattern of *14-3-3 σ* gene was highly affected by the originated cell type in the tumour.

*14-3-3 σ* is known to be one of the target genes of the tumour suppressor p53 protein. Alterations in the *p53* gene have been frequently observed in various human carcinomas (Hollstein *et al*, 1991), and are considered to be one of the most critical genetic changes in the process of carcinogenesis. However, unlike other carcinomas reported previously, ACC and MEC showed relatively low rates of alteration of *p53* in the present study. Kiyoshima *et al* (2001) and Papadaki *et al* (1996) also reported low frequency of the alteration of *p53* gene in ACC and MEC. Thus, the downregulation of 14-3-3 *σ* in ACC might not be due to the inactivation of p53 pathway, and inactivation of 14-3-3 *σ* by methylation may lead to impairment of some of the functions of p53 as a ‘gatekeeper’. Meanwhile, as shown in Table 1 and Figure 2, ACC3 showed downregulation of the 14-3-3 *σ* protein, but no methylation of the gene was detectable. However, if ACC3 showed mutation in *p53* gene (Table 1), then downregulation of 14-3-3 *σ* in ACC3 might be affected by the mutated p53.

Although most of ACC tissue simultaneously showed high methylation index of *14-3-3 σ* gene and downregulation of 14-3-3 *σ* protein, some cases did not show the correlation between the methylation index and expression level of 14-3-3 *σ*. The reason for this discrepancy was unclear at present, however, it might be due to contamination of the stromal cells for analysis or the presence of another pre- or post-transcriptional regulation of 14-3-3 *σ*. Recently, Urano *et al* (2002) demonstrated that Efp, a RING-finger-dependent ubiquitin ligase (E3) targeted proteolysis of 14-3-3 *σ*. In SGC cells, proteolysis of 14-3-3 *σ* by ubiquitin–proteasome pathway might contribute to regulate the level of 14-3-3 *σ* protein.

Using cultured-SGC cells, we found the downregulation of 14-3-3 *σ* in ACC cell lines with hemimethylation of the *14-3-3 σ* gene, but mutational analysis showed that ACCY cells carried the mutant type p53 at Tyr126Stop (data not shown). Therefore, the downregulation of 14-3-3 *σ* in ACCY cell lines was caused by the hemimethylation of *14-3-3 σ* gene and by the dysfunction of p53 pathway. Normal salivary gland cells, SG1, carried wild-type *p53* gene, and showed nonmethylation pattern of *14-3-3 σ* gene and high expression of *14-3-3 σ* mRNA and protein. DNA damage induced by the irradiation enhanced the expression of 14-3-3 *σ* protein in SG1 cells. These results indicated that expression of 14-3-3 *σ* in normal salivary gland cells was regulated by the activated p53 on the nonmethylated *14-3-3 σ* gene. HSG and TYS cells that were reported to carry mutant type p53 at Asn30Ser and Asp281His, respectively (Shinagawa *et al*, 2003). HSG and TYS cells showed different expression patterns of 14-3-3 *σ*. Shinagawa *et al* (2003) also reported that DNA-damaging chemotherapeutic drugs did not activate any reporter plasmids containing the promoter sequence of p53 target genes (*p21waf1*, *BAX*, *MDM2*, *p53AIP1*, or *PUMA*) in HSG cells, but activated only the p21waf1 promoter in TYS cells. Thus, mutated p53 in TYS cells, but not the mutated p53 in HSG cells, might affect the expression of 14-3-3 *σ*.

The *14-3-3 σ* gene has been reported to be a G2 checkpoint control gene. Since *14-3-3 σ (−/−)* cells are defective in their maintenance of G2 arrest, they enter M phase without repair of the aberrant chromosome structures and undergo cell death during mitosis (Chan *et al*, 1999). In the present experiment, apparent G2/M arrest was induced in normal salivary gland cells by irradiation; however, G2/M arrest was not induced by irradiation in the ACCY cells. Moreover, 14-3-3 *σ* was induced in normal salivary glands by irradiation, while 14-3-3 *σ* was not induced in ACCY cells. Although the affect of factors other than 14-3-3 *σ* could be considered, this fragility to the irradiation in ACC cells is in agreement with the clinical observations described previously (Simpson *et al*, 1984; Vikram *et al*, 1984; Miglianico *et al*, 1987; Garden *et al*, 1995; Parsons *et al*, 1996). These results indicate that DNA-damaging therapy might be more effective in cancers, such as ACC, with hypermethylation of *14-3-3 σ* gene than in cancers, such as MEC, without hypermethylation of *14-3-3 σ* gene.

In conclusion, our findings, that 14-3-3 *σ* is frequently downregulated in ACC, provide a new possible mechanism in understanding the mechanism of salivary gland carcinogenesis. Furthermore, our results also indicate that DNA-damaging therapy might be effective in SGCs with hypermethylation of *14-3-3 σ* gene. Downregulation of 14-3-3 *σ* could be useful as a predictive marker for the radiosensitivity of SGC, and the combination of 14-3-3 *σ* blockade and radiotherapy could constitute a new therapeutic approach for radioresistant SGC, particularly for MEC.
